# Antibacterial, Antiproliferative, and Immunomodulatory Activity of Silver Nanoparticles Synthesized with Fucans from the Alga *Dictyota mertensii*

**DOI:** 10.3390/nano8010006

**Published:** 2017-12-25

**Authors:** Marília Medeiros Fernandes-Negreiros, Raynara Iusk Araújo Machado, Fabiana Lima Bezerra, Maria Celeste Nunes Melo, Monique Gabriela Chagas Faustino Alves, Luciana Guimaraes Alves Filgueira, Marcelo Antonio Morgano, Edvaldo Silva Trindade, Leandro Silva Costa, Hugo Alexandre Oliveira Rocha

**Affiliations:** 1 Department of Biochemistry, Federal University of Rio Grande do Norte, Natal, Rio Grande do Norte 59078-970, Brazil; marilia_negreiros16@yahoo.com.br (M.M.F.-N.); raynara_yusk@hotmail.com (R.I.A.M.); monique.gabi@gmail.com (M.G.C.F.A.); lucianagalves@hotmail.com (L.G.A.F.); 2 Department of Microbiology and Parasitology, Federal University of Rio Grande do Norte, Natal, Rio Grande do Norte 59078-970, Brazil; bia@cb.ufrn.br (F.L.B.); celmelo@gmail.com (M.C.N.M.); 3 Food Science and Quality Center (CCQA), Institute of Food Technology (ITAL), Campinas 13070-178, Brazil; morgano@ital.sp.gov.br; 4 Departament of Cell Biology, Federal University of Parana, Curitiba 81531-980, Brazil; edstrindad@gmail.com; 5 Federal Institute of Education, Science and Technology of Rio Grande do Norte (IFRN), Ceara-Mirim, Rio Grande do Norte 59900-000, Brazil; leandro-silva-costa@hotmail.com

**Keywords:** Fucoidan, sulfated polysaccharides, melanoma, anti-bacterial agent, cytotoxicity, Dictyotales

## Abstract

In this study, we aimed to synthesize silver nanoparticles containing fucans from *Dictyota mertensii* (Martius) Kützing using an environmentally friendly method and to characterize their structure as well as antiproliferative, immunomodulatory, and antibacterial effects. Fucan-coated silver nanoparticles (FN) were characterized by Fourier-transform infrared analysis, dynamic light scattering, zeta potential, atomic force microscopy, energy dispersive X-ray spectroscopy, and inductively coupled plasma emission spectrometry. They were evaluated for their effect on cell viability, minimum inhibitory bactericidal concentration, and release of nitric oxide and cytokines. The FN were successfully synthesized using an environmentally friendly method. They were size-stable for 16 months, of a spherical shape, negative charge (−19.1 mV), and an average size of 103.3 ± 43 nm. They were able to inhibit the proliferation of the melanoma tumor cell line B16F10 (60%). In addition, they had immunomodulatory properties: they caused an up to 7000-fold increase in the release of nitric oxide and cytokines (IL-10; IL-6 and TNF-α) up to 7000 times. In addition, the FN showed inhibitory effect on Gram-positive and -negative bacteria, with MIC values of 50 µg/mL. Overall, the data showed that FN are nanoparticles with the potential to be used as antitumor, immunomodulatory, and antibacterial agents.

## 1. Introduction

Nanotechnology is a rapidly expanding area that focuses on improving compounds, making it an area of commercial interest. Several of its products such as nanoparticles have been demonstrated to be of interest within biotechnology due to their diverse application in several areas, silver nanoparticles (AgNPs) being of particular interest. Most of them show potent antibacterial [1], antiproliferative [2], and immunomodulatory [3,4] activities, which enable them to be used in several different areas such as in the cosmetic, pharmaceutical, and food industries [5]. Furthermore, AgNPs may be exploited to improve the treatments of cancers and immune disorders [6].

AgNPs are synthesized by various methods. However, many of these methods use substances that may be toxic to humans and/or to the environment. There is frequent concern about the lack of existing green chemistry processes for the development of nanoparticles in an environmentally friendly manner [7].

The green approach to nanoparticle synthesis involves the use of low cost, effective, and biocompatible materials that cause little damage to the environment and give rise to stable nanoparticles for several applications [8]. The “completely green” synthesis is a further advanced approach, where only water is used as a solvent and nontoxic agents such as carbohydrates are used as reducing agents [9].

Polysaccharides are known as low toxicity molecules. These compounds can be successfully used in the green synthesis of nanoparticles [6], because the process only requires the use of silver, water, and polysaccharides, without the use of stabilizers and catalysts. Several AgNPs synthesized using seaweed-sulfated polysaccharides (SSPs) showed cytotoxic activity against tumor cells, bacteria and other microorganisms [8]. SSPs have many pharmacological effects [9] that are related to the amount of sulfates as well as the position of these sulfate groups in the polysaccharide molecule [9]. In addition, each polysaccharide can present different applications [10].

Fucans are one of the most studied SSPs. They are sulfated polysaccharides based on sulfated α-L-fucose, synthesized by all brown seaweeds. The most commonly studied seaweeds belong to the Fucales, Laminariales, and Dictyotales orders [9]. There are several brown seaweed species of the Dictyotales order on the Brazilian coast, of which *Spatoglossum schröederi* is one of the most commonly studied. Recently, Amorim et al. [11] showed that AgNPs containing fucan from *S. schröederi* were more effective cytotoxic agents than the fucan alone, which indicated that the AgNPs could potentiate the fucan’s activities. Other Dictyotales, such as *Dictyota mertensii*, contain antioxidant, antiproliferative, and immunomodulatory SSPs [9,12]. However, there are no studies about AgNPs containing *D. mertensii* polysaccharides.

In the present study, we synthesized AgNPs containing sulfated polysaccharides from *Dictyota mertensii* (Martius) Kützing using an environmentally friendly method. The AgNPs’ physico-chemical characteristics, size, stability, composition, and shape were evaluated, as well as their antibacterial, antiproliferative, and immunomodulatory effects.

## 2. Results

### 2.1. Synthesis of Nanoparticles

**Figure 1:**
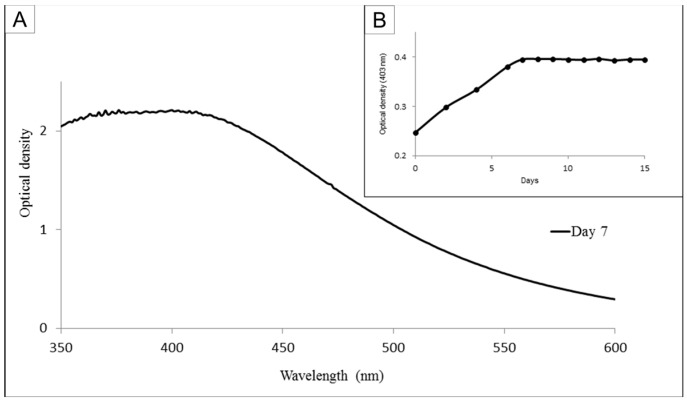
Light absorption profile of fucan-coated silver nanoparticles (FN) (**A**) Optical density in relation to the wavelength of 350 to 600 nm. (**B**) Optical density in light absorption ratio in 403 nm determined over 15 days.

Initially, we synthesized the AgNPs and using a spectrophotometer we performed a scan from 350 to 600 nm. We identified that 403 nm was the best wavelength to evaluate the formation of nanoparticles. Synthesis of the nanoparticles was performed over a period of 15 days (Figure 1). Each day an aliquot of the material was withdrawn and evaluated at 403 nm. We observed that optical density values increased up to the seventh day, and that the value did not change until the fifteenth day. The color change was an indication of the reduction of silver that occurs during nanoparticle synthesis. As the coloration did not increase after the seventh day, that day was chosen as the moment of fucan-coated silver nanoparticles (FN) synthesis with the highest yield, the yield of FN synthesis was 50.2 ± 1.8%.

### 2.2. Infrared Analyses

**Figure 2:**
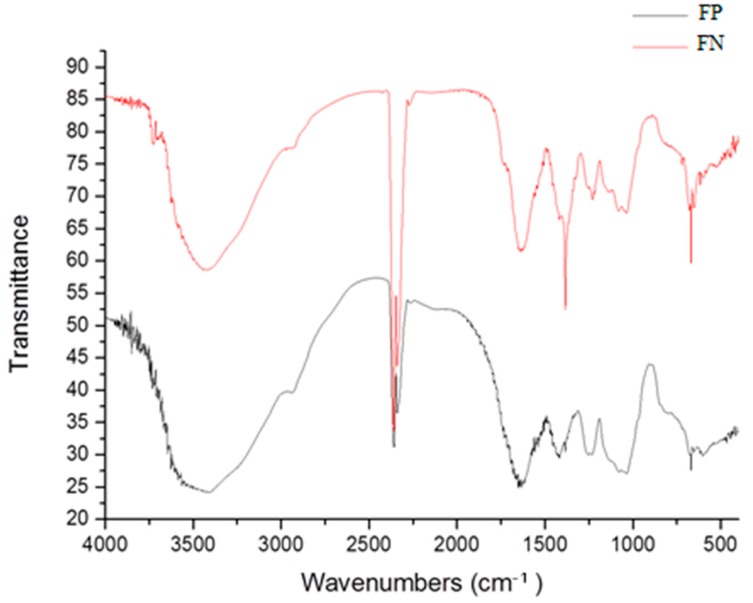
Fourier transform infrared spectroscopy (FTIR) spectra of fucan polysaccharide (FP) and fucan-coated silver nanoparticles (FN).

Infrared analyses were performed; the representative spectra of the samples are shown in Figure 2, and the main bands of these spectra are summarized in Table 1. In all samples, sulfated fucan polysaccharide (FP) characteristic absorption bands were observed in the regions: 3400, 3000, 1274, 1045, and 810–850 cm^−1^, with the exception of the region of 1400 cm^−1^, which is an indication of the silver and which was observed only in the FN spectra.

### 2.3. Characterization of FN

**Figure 3:**
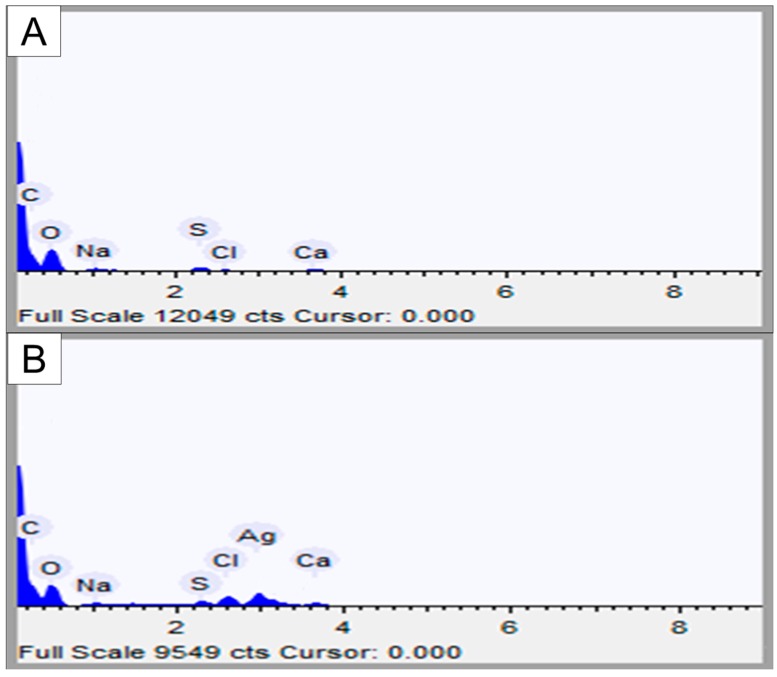
Energy dispersive X-ray spectroscopy (EDS) spectrum of FP (**A**) and FN (**B**).

Zeta potential analysis revealed that FP has a surface charge of −13.4 ± 3.6 mV and that FN have a surface charge of −19.1 ± 1.8 mV. Energy dispersive X-ray spectroscopy (EDS) analysis (Table 2 and Figure 3) indicated the elements on the surface of the sample. Oxygen, carbon, and sulfur were the main elements in both samples. The FN had less superficial oxygen and sulfur than FP. Additionally, FN had 0.96% of silver, as detected using an inductively coupled plasma optical emission spectrometry (ICP-OES) which did not occur in FP.

**Figure 4:**
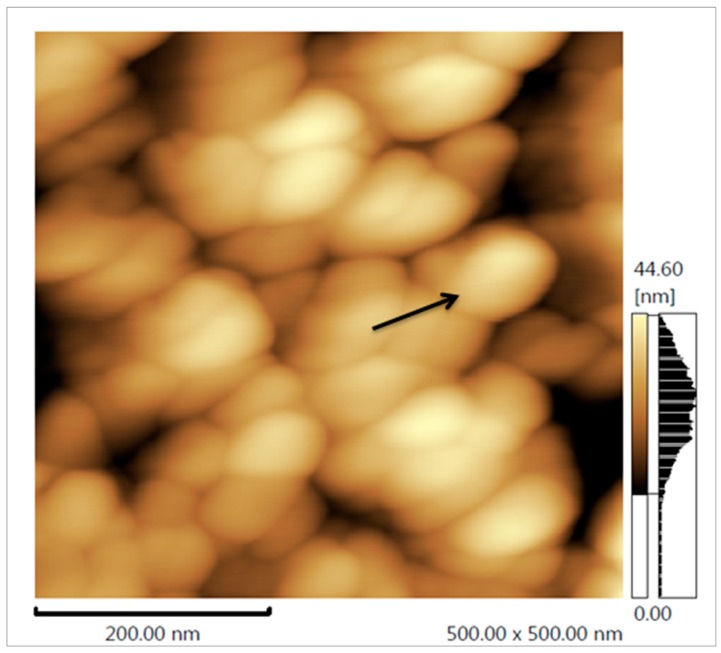
Atomic force microscopy (AFM) images of FN. The arrow points to one of the nanoparticles in the image.

With the use of dynamic light scattering (DLS), FN were characterized by mean size. In addition, FP had its size measured with the purpose of comparing it to the FN. The average size of FP was 1005.2 ± 51.1 nm. Figure 4 shows the histogram of particle size distribution recorded from the FN solution, as determined by DLS: the FN were evidently polydisperse. Furthermore, the average size of the FN was 103 ± 43 nm.

**Figure 5:**
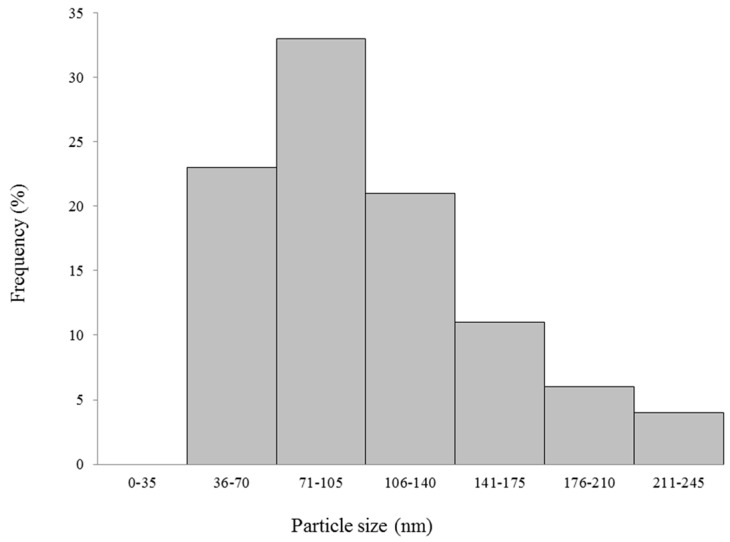
Size dispersion histogram obtained by dynamic light scattering (DLS).

Using Atomic Force Microscopy (AFM) we found that the FN had a round format (Figure 4). In addition, the diameter of the FN was also evaluated by AFM. As visible from the size dispersion histogram (Figure 5), the FN were polydisperse and the nanoparticles ranged from 50 to 100 nm.

In order to access the stability of the FN, their size were also periodically measured over 16 months by DLS, as described in Methods. On the day of the first measurement, the particle size was 103.33 ± 43 nm. This value did not change significantly until the last measurement. These data also indicate that the nanoparticles did not form aggregates during this time. In addition, the FN solution was dark brown, without any color change or visual aggregation up to the last day of observation.

### 2.4. MTT Assay

**Figure 6:**
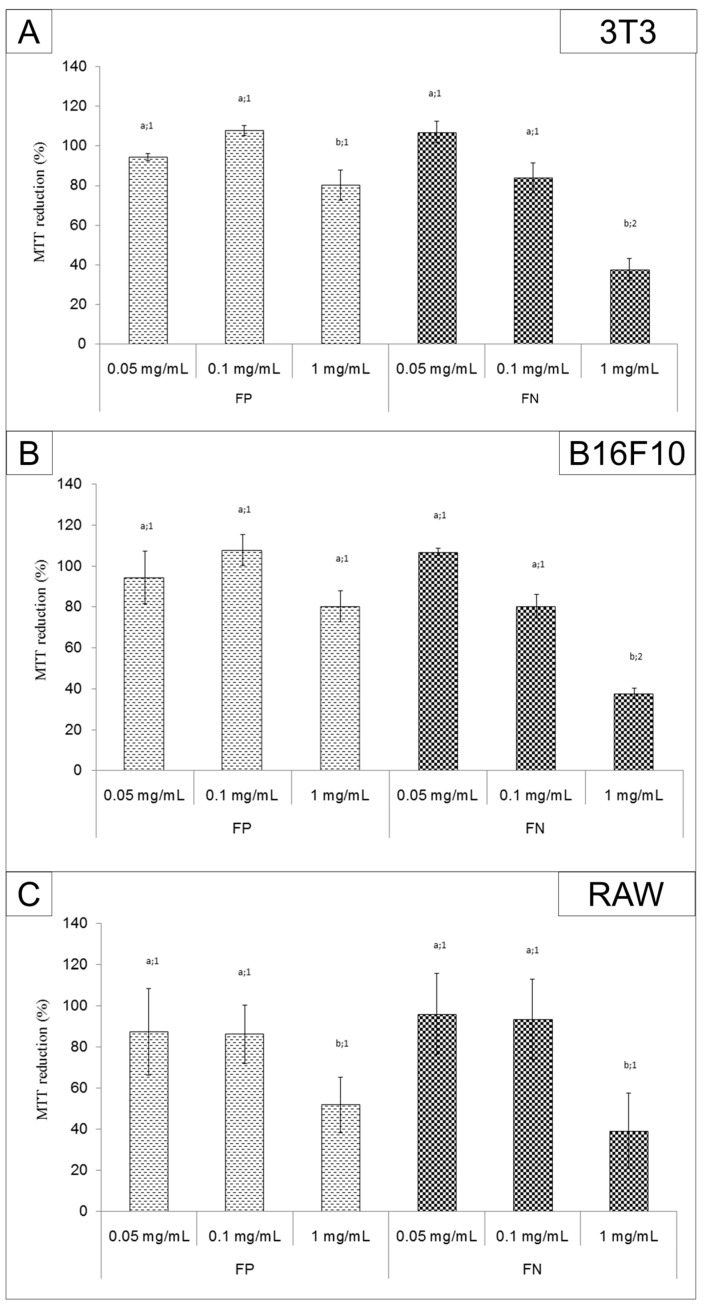
3-(4,5-dimethylthiazol-2-yl)-2,5-diphenyl-tetrazolium bromide (MTT) assay performed with (**A**) 3T3; (**B**) B16F10; and (**C**) RAW cells. The cells (1 × 10^4^) were exposed to Dulbecco’s modified Eagle’s medium (DMEM) containing the samples at concentration of 0.01, 0.05, 0.1, and 1 mg/mL for 24 h. Afterwards the cells’ ability to reduce MTT was measured as described in Methods. Different letters (a,b) represent the significant difference between FP or FN at different concentrations; different numbers (1,2) represent the significant difference between FP and FN in the same concentration by ANOVA followed by Newman-Keuls test (*p* < 0.05).

The ability of cells to reduce 3-(4,5-dimethylthiazol-2-yl)-2,5-diphenyl-tetrazolium bromide (MTT) in the presence of the samples is shown in Figure 6. 3T3 and RAW cells were affected by FP (Figure 6A,C). However, this effect was observed at a high concentration (1.0 mg/mL), and we observed a decreased ability to reduce MTT by around 20%. On the other hand, for all cells, the FN (1.0 mg/mL) decreased the ability to reduce MTT (Figure 6). In addition, the FN were a more potent inhibitor than FP, since, for 3T3, RAW, and B16F10 cell lines, the ability to reduce MTT decreased by about 60%.

### 2.5. Immunomodulatory Activities

First, an MTT assay was performed to identify the FP and FN concentrations that did not affect the ability of RAW cells to reduce MTT. The cells were incubated with FP or FN at concentration of 0.001, 0.01, 0.1, and 1.0 mg/mL for 24 h, and the MTT assay was performed as described in Methods. For both samples, 0.001 mg/mL was the concentration that did not affect the ability of RAW cells to reduce MTT. Therefore, this concentration of FP and FN was used for the following immunomodulation assays.

**Figure 7:**
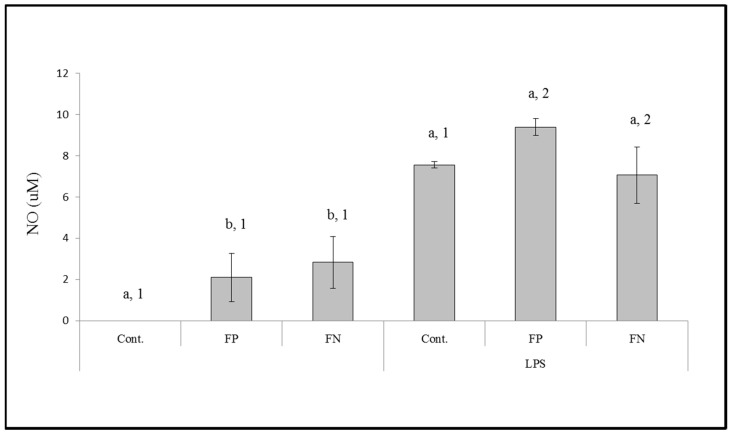
The effect of FP and FN on nitric oxide (NO) production by RAW 264.7 murine macrophages in the absence or presence of lipopolysaccharides (LPS). Different letters (a,b) represent significant difference between FP or FN at different concentrations; different numbers (1,2) represent the significant difference between FP and FN in the same concentration by ANOVA followed by Newman-Keuls test (*p* < 0.05).

Immunomodulatory activity of samples (at 0.001 mg/mL) was investigated by nitric oxide (NO) produced by macrophages (Figure 7). FP was able to stimulate NO synthesis, and this effect did not diminish when FP was in the nanoparticle form, since there was no significant difference between the amounts of NO synthesized in the presence of FP or FN. We did not observe a difference between the effects of FP and FN in the presence of lipopolysaccharides (LPS) either.

Immunomodulatory activity was also evaluated by cytokine production by macrophages incubated with FP or FN. Interleukin (IL) production was evaluated through T helper 1 (Th1; TNF-α, IL-2 and INF-γ), T helper 2 (Th2; IL-6, IL-10 and IL-4), and T helper 17 (Th17; IL-17A) responses (Table 3). Only the amounts of TNF-α, IL-6 and IL-10 were affected by the presence of FP or FN.

FP or FN alone were able to increase TNF-α (by about 12 times) and IL-6 (by more than 7000 times) release in comparison to the control group. In addition, when the cells were exposed to FP or FN in the presence of LPS, the amount of TNF-α released did not change. On the other hand, the amount of IL-6 increased compared to the amount of IL-6 released by the cells exposed to FP or FN without LPS. The amount of IL-10 released by cells exposed to FP or FN also increased in comparison to the control group. However, this increase was not as evident.

### 2.6. Antibacterial Activity

**Figure 8:**
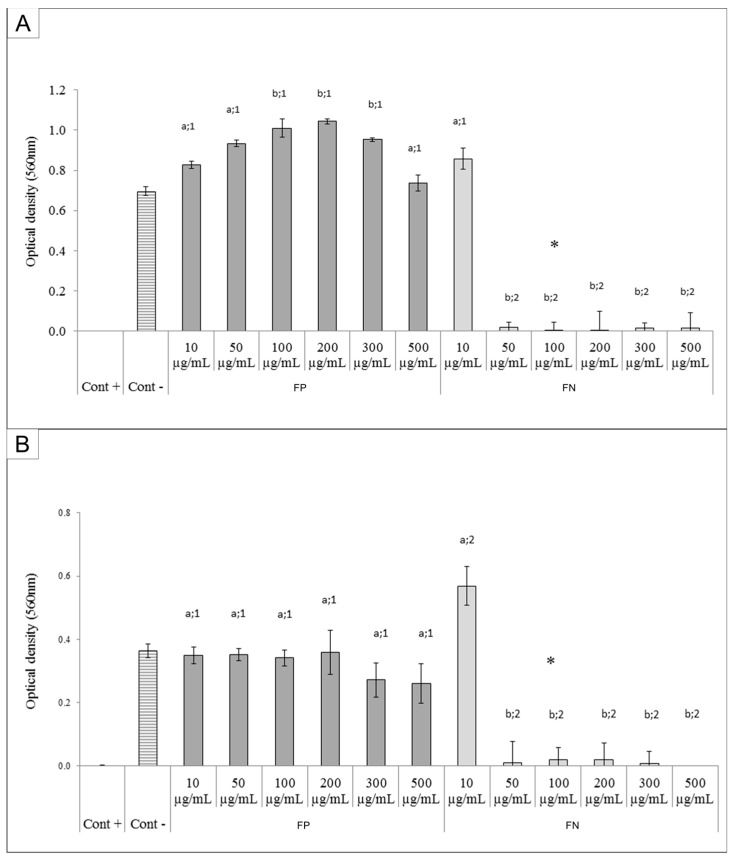
Minimum inhibitory concentration (MIC) activity and minimum bactericidal concentration (MBC) of FP from *D. mertensii* and FN for *E. coli* (**A**) and *S. aureus* (**B**). The samples were incubated at different concentrations (10, 50, 100, 200, 300, and 500 µg/mL). *—minimum bactericidal concentration; Cont. +, positive control (medium, bacterium, and antibiotic); Cont. −, negative control (medium and bacterium). Different letters (a,b) represent the significant difference between FP and FN at different concentrations; different numbers (1,2) represent the significant difference between FP and FN at the same concentration by ANOVA followed by Newman-Keuls test (*p* < 0.05).

*Escherichia coli* (Gram-negative bacterium) and *Staphylococcus aureus* (Gram-positive bacterium) were susceptible to FN solutions with a concentration of above 50 µg/mL. This concentration was able to stop the growth (identified by minimum inhibitory concentration—MIC), and the concentration of 100 µg/mL was able to kill these bacteria (identified by minimum bactericidal concentration—MBC). On the other hand, FP was not able to prevent bacterial growth (Figure 8A and B for *E. coli* and *S. aureus*, respectively).

## 3. Discussion

Our initial analyses were aimed at confirming that the FN are formed by sulfated polysaccharides, in this case fucans, and silver.

Infrared analyses confirmed the presence of polysaccharides. Bands at 3400 and 3000 cm^−1^ are characteristic of stretching of O–H and C–H, respectively, which indicates the presence of an organic component [4], and bands at 1000–1100 cm^−1^ indicate vibrations of C–O–C, characteristic of glycosidic bonds [13].

Infrared analyses also indicate that the polysaccharide is sulfated, due to the presence of the bands at 1274, 1045, and 810–850 cm^−1^, which are characteristic of asymmetric stretching of S=O, symmetric deformation of C–O–SO_3_, and flexural vibration of C–O–S, respectively [14]. These last two confirm the presence of sulfated polysaccharides (fucans) in FP and FN.

The specific absorption peak for silver in FN overlaps with the C–O–C stretching band at 1078 cm^−1^, and it cannot be separately identified. On the other hand, in the FN spectra, there is a band at 1400 cm^−1^ which was attributed to the presence of reduced silver [15]. Additionally, in FN spectra, there is a decrease (data not shown) in the size of the band at 1274 cm^−1^ in comparison to the FP spectra. This band is attributed to the asymmetric elongation of S=O, and its decrease indicates the association of the sulfate groups with the reduced silver [15]. Therefore, the infrared analysis indicates the binding of some sulfate groups to silver. The presence of silver nanoparticles was also confirmed by EDS analysis (Table 2). Taken together, these data allow us to affirm that we synthesized silver nanoparticles containing fucans from the alga *D. mertensii*.

The FN did not change their size for sixteen months, nor did they form aggregates during this period. These two parameters are used by several authors [16] to show the stability of nanoparticles. In our case, they showed that the FN remained stable for 16 months. Other nanoparticles synthesized thus far have not been stable for so long, although nanoparticles containing sulfated polysaccharides have been described as particles stable for several months [11,17]. An important feature is to be able to maintain the stability of nanoparticles and their surface charge. In the aqueous system, negative or positive charges in nanoparticles contribute to the repulsion between them, preventing their aggregation and instability [18]. Since FN have a negative charge, we attribute this characteristic as being responsible for their stability.

Comparing FP and FN sizes, we observed that FP is about 10 times bigger than the FN, probably because the fucans are in a configuration where they are more folded in the nanoparticle form. It has been reported that very small nanoparticles may exhibit high cytotoxicity compared to larger nanoparticles [16]. On the other hand, large nanoparticles are not easily absorbed by the tissues, making it difficult to act on their target tissue [6]. Thus, according to these studies, the sizes 20–200 nm are recommended for nanoparticles to be possibly applied in vivo. As our FN are within this size range, it is expected that they too will be suitable for in vivo applications.

Another important characteristic of nanoparticles is their shape. The FN are rounded, and this geometrical shape has already been described for several silver nanoparticles. In the case of polysaccharide nanoparticles, amorphous and other different geometrical shapes have not been identified so far. The exclusive rounded shape of polysaccharide nanoparticles is a positive feature, because this geometry is less cytotoxic compared to other forms, such as a triangular shape [19]. Thus, the FN are also expected to be less toxic to cells.

FP and FN cytotoxicity was evaluated by MTT assay. FP had low toxicity for 3T3, RAW, and B16F10 cells. On the other hand, the FN were more cytotoxic for cells than FP. Other studies have also shown that when fucans are used in the form of silver nanoparticles, they have a more potent activity than when they are used as isolated compounds [8,11]. The FN (1 mg/mL) inhibited the proliferation of the melanoma cell line B16F10 (~60%). A similar effect was observed by Kimura and colleagues [8], using a nanoparticle containing fucans from *Cladosiphon okamuranus*. However, these fucan nanoparticles were able to inhibit the proliferation of human osteosarcoma cells by only 80% when they were used at a concentration of 2 mg/mL. These authors also tested the fucan nanoparticles’ effect in vivo. They inoculated murine osteosarcoma LM8 tumor cells into the backs of mice, and treated the animals orally with 100 mg/kg/day of fucan nanoparticles. After 28 days, the authors showed that the fucan nanoparticles reduced the tumor volume from 1500 mm^3^ (positive control) to ~400 mm^3^. Hence, we expect the antiproliferative activity of FN to have an antitumor effect in vivo and will therefore evaluate the antitumor activity of FN in rats or mice in further studies.

We emphasize that, although the FN were toxic to 3T3 cells, all substances used as antitumor agents are also toxic to normal cells. Therefore, we cannot rule out the use of FN as an antitumor agent. Once the in vivo experiments are performed, the data we obtain may show us whether the toxicity of FN to tumor cells outweighs the toxic effect to normal cells.

Another way for a compound to have antitumor effects is to act as an immunomodulatory agent. Thus, we evaluated the effect of the FN on stimulating the release of NO and cytokines by macrophages (RAW cells). We identified that FP is able to stimulate the release of NO by macrophages and that this property had not diminished in the FN we synthesized. The mechanism by which fucans stimulate NO production, according to [20], is related to the presence and exposure of sulfate groups of these polysaccharides.

On the other hand, there are other forms of polysaccharides and polysaccharide nanoparticles that stimulate the immune system. We observed that FP and FN increased the release of TNF-α, IL-6, and IL-10; however, their effect was not identical for all cytokines. FP and FN were able to equally stimulate the release of IL-6 and IL-10, both in the presence and absence of LPS. However, when the cells were exposed to LPS and FN the release of TNF-α was higher than when cells were exposed to LPS and FP. On the other hand, in the absence of LPS, FP had a higher stimulatory effect on TNF-α release than FN. Other seaweed polysaccharides [21] and nanoparticles containing polysaccharides [3,4] were also able to stimulate the release of these same cytokines.

A positive point for the increase of production of cytokines is that these molecules are important in the protective response against harmful and excessive inflammatory action [22]. Overall, the FN appear to have the potential to be used as an immunomodulatory agent in vivo. Further in vivo studies will help to confirm this hypothesis.

The FN were able to inhibit the growth of both Gram-negative (*E. coli*) and Gram-positive (*S. aureus*) bacteria. Several studies have previously demonstrated the antibacterial effect of silver nanoparticles [23,24]. However, in these studies the AgNPs showed antibacterial activity at higher concentrations (0.8 to 1.6 mg/mL) than those used to achieve the same effect with the FN.

Some authors suggested the AgNPs act as silver ion reservoirs that permit the release of silver ions into bacteria [25]. In addition, it was reported that these silver ions promote membrane damage, increased cell permeability, and bacterial death [26]. As for the bactericidal effect of FN, we suggest that the bacterial metabolism degrades the polysaccharides present in the FN, allowing for a faster subsequent release of silver ions which then act as oxidizing agents in bacterial cell membranes.

Worldwide, different types of cancers are treated with chemotherapeutic agents. In addition, in many cases, patients also receive immunomodulatory agents to improve the treatment. Moreover, bacterial infections are common in patients exposed to these treatments. We have found a nanoparticle (FN) with antiproliferative, immunomodulatory, and antibacterial effects. Therefore, the FN have an enormous potential to be used as a multipotent agent in the fight against cancer, and we hope to prove this potential in vivo in the future.

## 4. Materials and Methods

### 4.1. Materials

Seaweed material—The seaweed *Dictyota mertensii* (Martius) Kützing was collected at Maracajaú beach (Rio Grande do Norte, Brazil, 5°40′99.40′′ S/35°31′08.86′′ W). The seaweed was taken to the Laboratory of Biotechnology of Natural Polymers (BIOPOL, Natal, RN, Brazil), Department of Biochemistry, at Universidade Federal do Rio Grande do Norte. There, it was washed, dried, crushed, and stored until use.

Bacterial strains—*Staphylococcus aureus* ATCC 25923 and *Escherichia coli* ATCC 25922 (natural strains from the American Type Culture Collection, Manassas, VA, USA) were used. They were thawed and activated. The activated bacteria were grown on 2 mL of brain heart infusion broth (HiMedia, Mumbai, India), at 37 °C for 24 h. Subsequently, they were transferred to the nutrient agar growth medium (HiMedia, Mumbai, India), and incubated at 37 °C for 24 h.

Eukaryotic cells—Mouse embryonic fibroblast cells (NIH/3T3 ATCC^®^ CRL-1658™-3T3, Manassas, VA, USA), *Mus musculus* skin melanoma cells (ATCC^®^ CRL 6475™-B16F10, Manassas, VA, USA), and *Mus musculus* macrophages (RAW 264.7 ATCC^®^ TIB-71^TM^, RAW, Manassas, VA, USA) were grown in Dulbecco’s modified Eagle’s medium (DMEM) with 10% of fetal bovine serum (FBS), 10 mg/mL streptomycin, and 10,000 IU penicillin.

### 4.2. Fucan Polysaccharides (FP) Extraction

Seaweed Material was proteolyzed in a 0.25 M NaCl solution containing a mixture of alkaline proteases (Prozyn, PROLAV 750, Prozyn Biosolutions, São Paulo, SP, Brazil) at 60 °C, and pH 8. After 16 h of incubation, the mixture was filtered and centrifuged at 9500× *g*, at 4 °C, for 15 min [12]. In order to precipitate the fucan polysaccharides (FP), two volumes of methanol were added into the supernatant and the material was kept at 4 °C for 12 h. The FP were separated from the solution by centrifugation (9500× *g*, 4 °C, 15 min), dried at low pressure, triturated, and stored for later analysis.

### 4.3. Nanoparticle Synthesis

Nanoparticle were synthesized according to a method previously described [11]. Briefly, solutions of FP (10 mg/mL) were added to a silver nitrate solution (1 mM in ultrapure water) at a proportion of 1:9 and left to rest. In order to determine the day on which the largest amount of nanoparticles could be obtained, the absorbance of the nanoparticle solutions was monitored from 350 to 600 nm for 15 days. Figure 1B shows the wavenumber obtained at 403 nm at each day. The material obtained on day 7 was centrifuged twice (11,500× *g*, 20 min, 4 °C), suspended in ultrapure water and lyophilized. The precipitate obtained was of fucan-coated silver nanoparticles (FN). In order to determine the yield of FN synthesis, the amount of FP used in this synthesis was established as 100%.

### 4.4. Nanoparticle Characterization

Fourier transform infrared spectroscopy analysis was performed by pressing 5 mg of each sample with 10 mg of KBr, and the resulting material was analyzed with a spectrometer Shimadzu FTIR-8400S (Kyoto, Japan). Thirty scans were performed for each sample, in the range between 400 to 4000 cm^−1^ at room temperature and the correction of the baseline was calculated using an IRSolution software version 1.60SU1 (Shimadzu, Kyoto, Japan). The dynamic light scattering (DLS) measurement and the zeta potentials of the samples were performed at 25 °C on a Zeta Potential Analyzer (Brookhaven, New York, NY, USA). Briefly, FN suspensions (0.5 mg/mL) were analyzed in three independent experiments (*n* = 10), and the reported values correspond to mean ± SD.

The FN stability was evaluated by DLS. Briefly, an FN suspension (0.5 mg/mL) was prepared and stored in the dark at 4 °C for 16 months. Measurements were made every thirty days as described earlier. In addition, we also observed color change, visual aggregation, and precipitate formation.

Atomic force microscopy (AFM) and energy dispersive X-ray spectroscopy (EDS) were performed by a Scanning Probe Microscope (SPM) 9700 (Shimadzu, Kyoto, Japan) and a Scanning Electron Microscope TM-3000 (Hitachi, Kyoto, Japan), respectively, at the Scanning Electron Microscopy Laboratory (LABMEV), in the Department of Engineering Materials (DEMat), of Universidade Federal do Rio Grande do Norte (UFRN). At least three images were taken in different fields. All images were evaluated for the shape of the samples.

One drop of the FN suspension (0.5 mg/mL) was dried on a glass cover slip and submitted to AFM analysis. EDS analyses were made with dry FN that was dropped onto carbon tape.

FN digestion was performed. Analytical scales were used to weigh 100 mg of sample in teflon cups (200 mg of sample in total, 100 mg per cup), to which 7 mL of 65% nitric acid purified by sub-boiling distillation (Berghof, Eningen, Germany) and 1 mL of 30% hydrogen peroxide (Merck, Darmstadt, Germany) were added. The digestion cups were subsequently closed and digestion was performed in a microwave digester (Start D, Milestone, Italy) using six stages and a power of 1100 W: (1) 5 min at 70 °C; (2) 2 min at 70 °C; (3) 3 min at 120 °C; (4) 2 min at 120 °C; (5) 10 min at 170 °C; (6) 15 min at 170 °C and lastly 30 min of ventilation before the removal of the rotor from the microwave. Deionized water was added to the digested content to fill up to 15 mL and filtered through a 0.45 µm membrane. The analyses were conducted in duplicate and analytical blanks were performed by conducting the procedure in the absence of the sample.

The silver content of the digested FN was quantified using an inductively coupled plasma optical emission spectrometry (ICP OES 5100 VDV, Agilent Technologies, Tokyo, Japan) in axial view, equipped with a radio-frequency source (RF) of 27 MHz, using a simultaneous optical detector, a peristaltic pump, a double pass cyclonic spray chamber, a 1.8 mm quartz torch, and a SeaSpray glass nebulizer. The gas used in the system was plasma liquid argon with a purity of 99.996% (White Martins, SP, Brazil). The ICP OES equipment operated under the following conditions: plasma power, 1.5 kW; argon flow, 12.0 L/min; auxiliary argon flow, 1.0 L/min; nebulization flow, 0.70 L/min; stabilization and reading time, 15 s; wavelength, 328.068 nm; number of replicates, three.

The analytical curve for silver was prepared by diluting 1000 mg/L of the reference standard solution (Merck, Darmstadt, Germany) to a 2.5 to 100 µg/L range (r = 0.9999) in a solution of 5% (*v/v*) nitric acid prepared from 65% acid distilled in a sub-boiling system (Distill acid; Berghof, Eningen, Germany). Serial dilutions were prepared for sample measurement: (i) 0.1 mL/10 mL and (ii) 0.2 mL/10 mL.

### 4.5. Antibacterial Activity

In vitro antibacterial activity was evaluated using minimum inhibitory concentration (MIC) and minimum bactericidal concentration (MBC) methods.

#### 4.5.1. Minimum Inhibitory Concentration (MIC) Test

The MIC test was performed by the broth microdilution method according to the Clinical and Laboratory Standards Institute (CLSI, 2012). Initially, samples were dissolved in different concentrations (500, 300, 200, 100, 50, 20, and 10 µg/mL) of Mueller Hinton medium (HiMedia) and were plated onto 96 well plates. In parallel, the activated bacteria were standardized to a turbidity equivalent to 0.5 on the McFarland scale, according to CLSI (2012), and then 10 μL of bacterial inocula was added to the wells of the microplates containing samples (the inoculum concentration per well was 5 × 10^5^ CFU/mL).

For the positive control (wells with bacterium and antibiotic), the antibiotic gentamicin was used, and for the negative control (wells with bacteria, but without antibiotic), only saline and medium solution was added. Bacterial growth was verified after 24 h of incubation at 35 °C, by optical density at 560 nm. The MIC of each sample was interpreted as the lowest concentration that was able to completely prevent bacterial growth. The activity was exposed after discarding the whites of all samples. All assays were carried out in triplicate.

#### 4.5.2. Minimum Bactericidal Concentration (MBC) Test

To determine the MBC, dilutions of bacterial suspensions apart from the MIC were prepared as described under the MIC test and transferred to nutrient agar (HiMedia). The plates were incubated at 35 °C for 24 and 48 h. The MBC was defined as the lowest concentration of samples (FN/FP) which yielded 99.9% killing.

### 4.6. Antiproliferative Activity

For the tests, 0.5 × 10^4^ cells were grown in 96-well plates with DMEM medium containing the samples in concentrations of 0.01, 0.05, 0.1, and 1 mg/mL for 24 h (each concentration in triplicate). Cell viability was determined by the colorimetric test of 3-(4,5-dimethylthiazol-2-yl)-2,5-diphenyl-tetrazolium bromide (MTT) as described earlier [11].

### 4.7. Synthesis of Fucan-Coated Silver Nanoparticle (FN)

Fucan-coated silver nanoparticle (FN) were obtained using a green synthesis procedure similar to that described by Dipankar and Murugan [24], using fucan as a bioreductor. Synthesis of the FN was performed by the addition of a 1.0 mM solution of silver nitrate to a solution of fucan (10 mg/mL) (1:9 *v/v*). The solution was homogenized in a round bottom flask, previously protected from light with aluminum foil, and subjected to stirring. After 24 h, the suspension was centrifuged at 10,000× *g* for 15 min at 25 °C. The precipitate was collected, lyophilized, and kept protected from light in a desiccator.

### 4.8. Immunomodulatory Activity

Immunomodulatory activity was evaluated by nitric oxide (NO) and cytokine production assays on macrophages incubated with the samples.

For both tests, *Mus musculus* macrophages (RAW 264.7 ATCC^®^ TIB-71TM) were cultured in 24 well plates (3 × 10^5^ cells/well) with DMEM containing 10% FBS, 10 mg/mL streptomycin, and 10,000 IU penicillin.

The MTT assay was performed with RAW cells incubated with a different amount of sample (0.001, 0.01, 0.1, and 1.0 mg/mL), as described above.

For the NO test, after 24 h of incubation for cell adhesion, the medium was removed and a new medium containing 0.001 mg/mL of FP or the FN was added. After 24 h, the supernatant (100 μL) from each well was removed and added to 100 μL of Griess reagent (1% sulfanilamide in 5% phosphoric acid and 0.1% naphthylethylenediamine dihydrochloride in water). The presence of nitric oxide was observed by color change, which was monitored using a microplate spectrophotometer (Epoch; BioTek, Winooski, VT, USA) at 560 nm. For positive control, we used the medium from RAW cells exposed to 2 mg/mL of lipopolysaccharides (LPS; Sigma-Aldrich, St. Luis, MO, USA), and the negative control was the medium from the cells not exposed to LPS or the samples. Sodium nitrite was used as the standard.

In the cytokine production assay, after 24 h cell adhesion, the medium was removed and a new medium containing 0.001 mg/mL FP or FN was added. After 24 h, the amount of interleukin-2 (IL-2), interleukin-4 (IL-4), interleukin-6 (IL-6), interferon-γ (INF-γ), tumor necrosis factor (TNF-α), interleukin-17A (IL-17A), and interleukin-10 (IL-10) in the cellular medium were determined according to the procedures indicated by Cytometric Bead Array (CBA) Mouse Th1/Th2/Th17 Cytokine (BD) kit (BD Biosciences, San Diego, CA, USA) using a flow cytometer (FACSCANTO II, BD Bioscience, Franklin Lakes, NJ, USA).

### 4.9. Statistical Analysis

Data were expressed as mean values ± standard deviations. The data in triplicates were analyzed by analysis of variance (ANOVA). The Student Newman–Keuls test (*p* < 0.05) was used to determine significant differences among the samples tested using the program GraphPadPrism version 5.0, 2014 (GraphPad, La Jolla, CA, USA).

## 5. Conclusions

FN were successfully synthesized from FP using an environmentally friendly method. These nanoparticles were stable for a long period and were of a spherical shape, negative charge, and reduced size. The FN were able to inhibit the proliferation of the B16F10 tumor cell line. They also had immunomodulatory properties, since they stimulated the release of cytokines (IL-10, IL-6, and TNF-α) and NO. In addition, the FN also had antibacterial effect against Gram-negative and Gram-positive bacteria.

## Figures and Tables

**Table 1:** Infrared spectra (peak positions) of fucan polysaccharide (FP) and fucan-coated silver nanoparticles (FN).

Chemical Group	O–H	C–H	C–O–C	S=O	C–O–SO_3_	C–O–S	“Silver Reduction”
FP	3418.7	2941.5	1080.1	1228.8	1036.1	810.8	-
FN	3429.1	2942.0	1082.3	1130.4	1035.8	811.0	1383.8

**Table 2:** Energy dispersive X-ray spectroscopy (EDS) of fucan polysaccharide (FP) and fucan-coated silver nanoparticles (FN).

	Sulfur (%)	Oxygen (%)	Carbon (%)	Silver (%)
FP	0.95	54.82	41.69	0
FN	0.54	33.44	62.16	1.45

**Table 3:** Results of the cytokine production assay (identification of TNF-α, IL-2, INF-γ, IL-6, IL-10, IL-4, and IL-17A) on macrophages incubated with 0.001 mg/mL FP or FN.

*	LPS	TNF-α	IL-2	INF-γ	IL-6	IL-10	IL-4	IL-17A
Cont.	-	598.7	0	0	0	0	0	0
	+	6999.0	0	0	9286.6	0	0	0
FP	-	7607.4	0	0	7815.4	0	0	0
	+	7072.1	0	0	11,362.7	15.2	0	0
FN	-	7296.4	0	0	7522.0	0	0	0
	+	7768.28	0	0	11,695.6	15.2	0	0

* The standard deviations for all of the data were less than 5%.
